# New Scenarios in Heart Transplantation and Persistency of SARS-CoV-2 (Case Report)

**DOI:** 10.3390/life13071551

**Published:** 2023-07-13

**Authors:** Lubov Mitrofanova, Igor Makarov, Andrey Gorshkov, Olga Vorobeva, Maria Simonenko, Anna Starshinova, Dmitry Kudlay, Tatiana Karonova

**Affiliations:** 1 Almazov National Medical Research Centre, St. Petersburg 197341, Russia; mitrofanova_lb@almazovcentre.ru (L.M.); gorshkov_an@almazovcentre.ru (A.G.); vorobeva_om@almazovcentre.ru (O.V.); simonenko_ma@almazovcentre.ru (M.S.); karonova_tl@almazovcentre.ru (T.K.); 2 Smorodintsev Research Institute of Influenza, St. Petersburg 197376, Russia; 3 Department of Pharmacology, I.M. Sechenov First Moscow State Medical University, Moscow 119992, Russia; d624254@gmail.com; 4 Institute of Immunology FMBA of Russia, Moscow 115478, Russia

**Keywords:** SARS-CoV-2 infection, immunohistochemical and ultrastructural myocardial studies, heart transplantation, virus transmission

## Abstract

Heart transplantation is a treatment of choice for patients with severe heart failure. Infection transmission from a donor to a recipient remains a prominent problem in organ transplantation. However, the risk of SARS-CoV-2 transmission in nonlung organ transplantation is still unclear. In this article we presented a case of a 28-year-old pregnant woman who developed heart failure soon after recovery from a SARS-CoV-2 infection in the third trimester of gestation. In the postpartum period, the heart disease worsened and the patient required cardiac transplantation. We examined the recipient’s heart and made a diagnosis of left ventricular noncompaction cardiomyopathy. Immunohistochemical analysis showed SARS-CoV-2 antigen expression in the donor’s heart before transplantation, and after the transplantation, an endomyocardial biopsy was taken. Moreover, an ultrastructural assessment of the endomyocardial specimen revealed endothelial and pericyte injury and a single particle on the surface of the endothelium consistent with SARS-CoV-2 viral particles. Recent findings in the literature associated these damages with SARS-CoV-2 infection. The present study describes the rare case of SARS-CoV-2 transmission from donor to postpartum recipient through a heart transplant and demonstrates the importance of endomyocardial biopsy before and after heart transplantation.

## 1. Introduction

The first heart transplant was performed in 1967. Since then, it has remained the definitive treatment for patients with end-stage heart failure (HF). Patients with pre-existing heart conditions are more susceptible to SARS-CoV-2 infection and are at a higher risk of morbidity and mortality [1]. Therefore, it is not surprising that during the SARS-CoV-2 pandemic, there was an increasing number of patients with organ transplants who suffered from COVID-19 [2]. Since these patients received immunosuppression therapy, the course of the disease was more severe [3,4].

## 2. Case Presentation

The patient, a 28-year-old pregnant woman at the 34th week of gestation, was diagnosed with SARS-CoV-2 infection and had bilateral pneumonia. Ten days after recovery from the viral infection, the patient started to experience HF symptoms: shortness of breath; swelling in the legs, ankles, and feet; and heart palpitations. On clinical examination, the following data were determined: bilateral interstitial pneumonia and hydrothorax; sinus tachycardia and nonspecific T-wave; significant chamber dilation and signs of left ventricular (LV) hypertrophy; LV ejection fraction was 37% (Table 1).

At the 39th week of gestation, the healthy newborn was delivered via cesarean section.

In the post-partum period, the patient still had symptoms of HF: dyspnea upon exertion, edema of ankles and feet, a high level of N-terminal pro-B-type natriuretic peptide (NT-proBNP) at 3506 pg/mL, and a progressive decline in LV ejection fraction to 17%. Concomitant infection resulted in an aggravation of HF symptoms, e.g., recurrent atrial flutter.

Because of the end-stage HF, the patient was eventually transferred to an extracorporeal membrane oxygenation (ECMO) facility. To define the origin of HF, a right ventricular endomyocardial biopsy (EMB) was carried out.

Myocarditis, cardiomyopathy, ischaemic heart disease, arterial hypertension, heart defects, pulmonary embolism, and other causes were excluded as causes of heart failure.

Upon histological examination of EMB samples, moderate myocyte degeneration was observed. Immunohistochemical studies identified the following cell types: CD3+ activated T lymphocytes (5 per 1 mm^2^), CD68+ macrophages (16 per 1 mm^2^), enterovirus-VP1-positive cardiomyocytes (5–20%), and HHV-6-positive lymphocytes. The cytomegalovirus antigen was not detected. Endothelial activation was confirmed by the expression of VWF, VEGF, and C1q, but HLA-DR was negative. Thus, we concluded the absence of myocarditis signs in EMB specimens.

**Figure 1:**
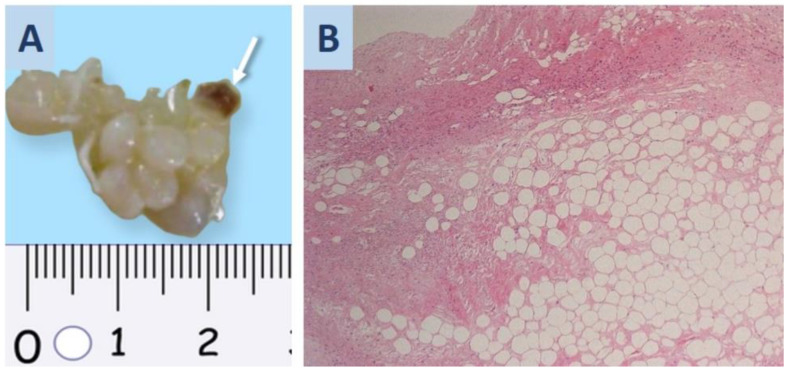
Atrial tumor surgical material was found by cardiac surgeons in a donor heart during transplantation and sent to the pathology department for urgent histological examination. (**A**) Polypoid fibrolipoma in donor’s left atrium; the arrow indicates the myocardial site. (**B**) Fibrous and adipose tissue structure of fibrolipoma; hematoxylin-eosin staining; ×100.

Orthotopic heart transplantation (HTX) was carried out after 18 days of ECMO therapy. For HTX, we used the bicaval technique under extracorporeal circulation, and myocardial protection was provided with Custodiol cardioplegia. According to our local protocol, all donors and recipients were examined to rule out COVID-19 before the HTX: a swab PCR test and thoracic CT scan were performed in both of them prior to surgery. During the surgery, a polypoid white formation of size 2 × 0.7 cm was found in the donor’s atrium. It was removed together with a 0.4 × 0.3 cm fragment of the myocardium. After histological examination, this case was diagnosed with polypoid fibrolipoma (Figure 1A,B).

**Figure 2:**
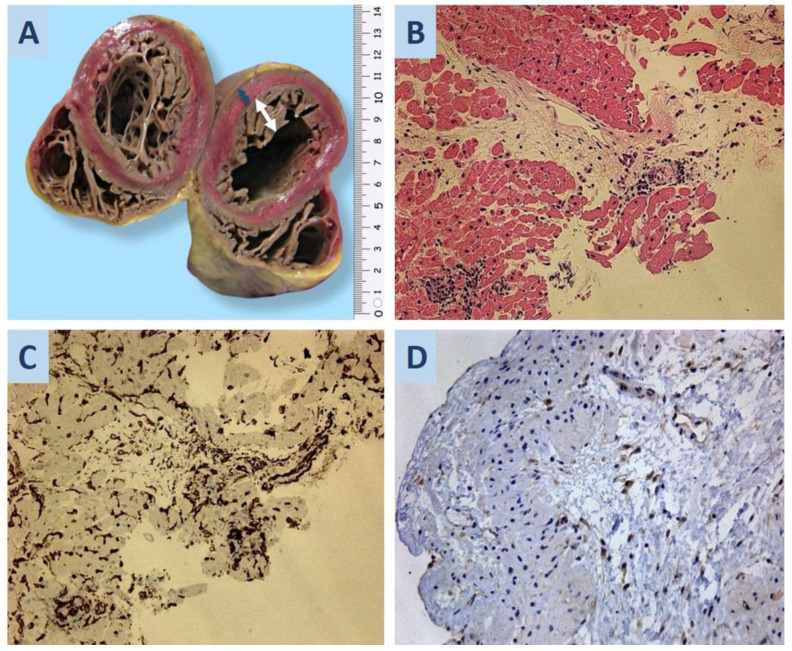
(**A**) Noncompact myocardium of the left ventricle: the blue arrow shows the thickness of the compact layer (0.6 cm), and the white arrow shows the non-compact layer (1.6 cm). (**B**) Second EMB of the donor heart: a visible infiltrative vasculitis with macrophages in the lumen of blood vessels; hematoxylin-eosin staining; ×200. (**C**) Immunohistochemical staining shows CD34 expression in large, polymorphic endothelial cells; ×200. (**D**) SARS-CoV-2 S-protein expression in the cytoplasm of large, polymorphic endothelial cells; ×200.

The explanted heart from the recipient was globular, weighed 387 g, and had a size of 11.0 × 11.0 × 6.0 cm. Coronary arteries were free of stenosis. As seen in the figure (Figure 2A), the ventricular cavities were affected by excessive trabeculation, and mural thrombi were prominent in the intertrabecular spaces. The thickness of the apical and mid-ventricular regions of the posterior and lateral walls of LV was 0.6 cm. The thickness of the trabeculated myocardium was 1.6 cm. In the cross-section, the myocardium was homogeneous and was brown in color.

Histologically, the myocardium showed signs of parietal thromboendocarditis of 2–3 weeks with fibrin deposition, lymphocytic infiltration, and granulation tissue. Microscopic examination of 15 sections of LV free wall found two samples with lymphocytic infiltrates (10 and 23 cells per 1 mm^2^). The report diagnosed LV noncompaction cardiomyopathy, acute focal myocarditis, and parietal thromboendocarditis of LV. The latter is possibly part of post-COVID-19 syndrome. However, immunohistochemical studies did not reveal SARS-CoV-2 antigen expression.

After heart transplantation, the recipient was managed with triple-drug immunosuppression: tacrolimus, mycophenolic acid, and steroids. A few weeks before HTX, she was treated for multi-drug-resistant bacterial pneumonia with different schemes of I.V. antibacterial treatment. It was decided not to prescribe induction therapy, considering her recent recovery from infectious complications, the duration of ECMO support, and initiation of triple-drug immunosuppression after HTX to minimize the risk for infection development.

Eight days post-HTX, the patient had the first EMB, and its examination detected the following rejection grade of the cardiac graft: acute cellular rejection (ACR (0R)) and antibody-mediated rejection (pAMR2 (I+, H+)). Twenty days post-HTX, signs of humoral rejection (pAMR2 (I+, H+)) persisted in the second EMB sample. Pathomorphological analysis of both EMB samples (8 and 20 days) additionally found hypertrophic, polymorphic, perivascular cells with large nuclei (Figure 2B–D). We described these cells as activated endothelial cells because of CD34 expression, where the SARS-CoV-2 antigen was also positive. However, ddPCR did not confirm the presence of SARS-CoV-2 RNA in the EMB sample. An ultrastructural study was performed only for the second EMB (20 days post-HTX).

**Figure 3:**
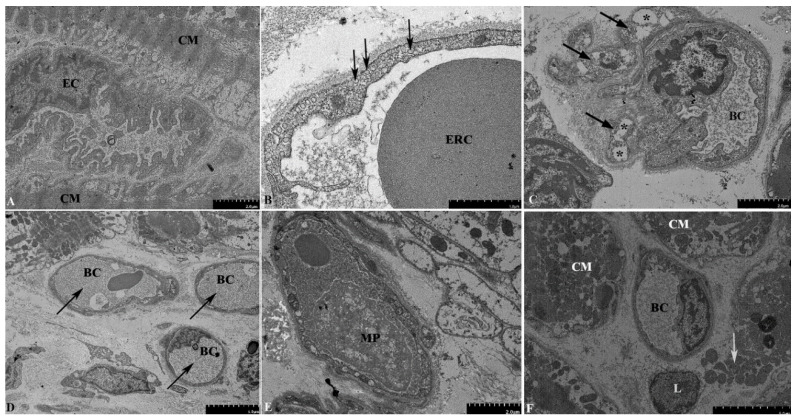
Electron microscopy examination of the second EMB of the donor heart. (**A**) Irregularly shaped endothelial cells, with an indented luminal surface and irregular nuclei protruding into the lumen of the blood capillary. (**B**) A large number of vesicles in the cytoplasm of endothelial cells (arrows). (**C**) Pericyte processes (arrows) in close contact with the blood capillary. The processes contain vacuoles up to 1 µm in size (asterisks). (**D**) Obstruction of the capillary lumens by a fibrillar material of medium electron density, presumably fibrin (arrows). (**E**) Macrophages containing multiple phagocytic vacuoles in the capillary lumen. (**F**) Necrosis of cardiomyocytes, with a rupture of the plasma membrane and the release of intracellular organelles into the extracellular space (arrows). Abbreviations: EC—endothelial cell, CM—cardiomyocyte, BC—blood capillary, ERC—erythrocyte, MP—macrophage, L—lymphocyte.

We found clear signs of ultrastructural remodeling of blood capillaries. Many endothelial cells were irregularly shaped, with an uneven and indented luminal surface and large, irregular nuclei protruding into the lumen of the blood capillary (Figure 3A). The cytoplasm was packed with numerous membrane vesicles, both plasma membrane-bound and unbound, thus indicating activation of the endothelium (Figure 3B). Pericyte processes were in close contact with the capillaries. As endothelial cells, pericyte processes contained abundant vesicles and vacuoles up to 1 µm in size (Figure 3C).

Cytoplasmic vacuolization can be transient or irreversible, and the latter is usually observed in cells infected with bacterial or viral agents. The lumen of some sections of the capillaries was completely obstructed by fibrillar material of moderate electron density, presumably fibrin (Figure 3D). In some of the capillaries, macrophages containing multiple phagocytic vacuoles were detected (Figure 3E). While most of the cardiomyocytes retained normal ultrastructural organization, some of them showed signs of necrotic death, e.g., rupture of the plasma membrane and release of intracellular organelles (Figure 3F).

**Figure 4:**
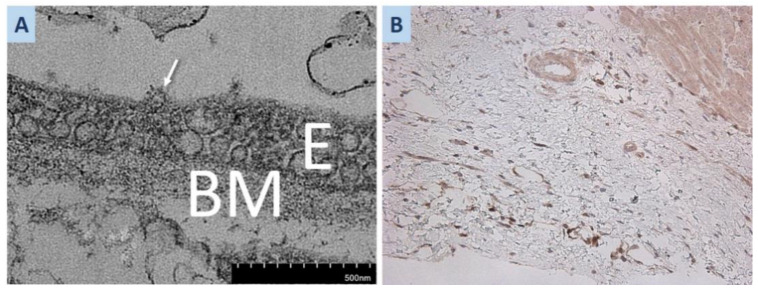
(**A**) Second EMB of the donor heart. Round 100 nm particles with clear signs of morphological similarity to the SARS-CoV2 virion (arrow), localized at the apical surface of the endothelium. (**B**) SARS-CoV-2 S-protein expression in the cytoplasm of endothelial cells of the polypoid fibrolipoma myocardial site; ×100. Abbreviations: E—endothelium, BM—basal membrane.

In one section, we found a 100 nm round particle with clear signs of morphological similarity to the SARS-CoV2 virion, being associated with the apical surface of the endothelial cell (Figure 4A). Some of the aforementioned ultrastructural changes found in EMB are known to be histopathological features of AMR [5].

Thirty-four days post-HTX, we performed the third EMB for recurrent assessment of the rejection grade. They were defined as follows: ACR (1R/1A) and pAMR0.

In the postoperative period, LV function was preserved, and sinus rhythm was maintained during the follow-up period.

Additionally, we performed a retrospective immunohistochemical study of polypoid fibrolipoma from the donor heart to determine the expression of SARS-CoV-2 antigens, which would define the donor to be the carrier of SARS-CoV-2. The SARS-CoV-2 antigen was present only in the donor heart (Figure 4B). Examination in the recipient heart did not identify histological signs of SARS-CoV-2. There were no diagnosed COVID-19 cases in patients or healthcare practitioners who were in contact with recipient while she was waiting for transplantation. After surgery, the recipient stayed in the individual patient room and advanced sanitary conditions were organized based on the hospital’s protocol. These observations support that she was infected with SARS-CoV-2 from the heart transplant donor.

## 3. Discussion

According to the reviews summarizing the aspects of COVID-19 in transplant patients, the median intervals from transplantation to diagnosis were from 26 months to 10 years [6,7].

Cases of SARS-CoV-2 infection in the peritransplant period are scarce. Qin et al. described a liver transplant recipient presenting with perioperative COVID-19 that was not detected upon admission [8].

Proven donor-to-recipient transmission of SARS-CoV-2 in heart transplantations is rarely documented. Several studies have described successful transplantations of non-pulmonary organs from SARS-CoV-2-infected donors to infected and uninfected recipients [9,10,11,12,13,14]. Moreover, transplantation from donors infected with SARS-CoV-2 has also not resulted in the infection of the recipient [15,16,17,18,19]. On the other hand, proven transmission of SARS-CoV-2 in lung transplantation has been reported [20,21].

In both cases, the donors were asymptomatic and had negative nasopharyngeal smear PCR test results, which is indeed not sufficient to rule out SARS-CoV-2 in internal organs. Certainly, in such cases, additional serological testing for specific SARS-CoV-2 immunoglobulins, PCR of smears from deeper sections, and a morphological examination of donor biopsy material are appropriate. However, it is not always possible due to time constraints and the severity of the recipient’s condition, as in our case. In any case, the risk of SARS-CoV-2 transmission through organ donation remains underestimated.

Our case is exciting because the trigger for the development of CHF in a pregnant patient with noncompact LV myocardium was COVID-19 with myocarditis and thromboendocarditis, leading her to TC. But at the time of EMB before TC, we detected only markers of humoral immunity activation and endothelial cell activation. These symptoms may be related to post-COVID-19 syndrome. Naturally, we cannot completely rule out a recurrence of COVID-19 infection in the recipient, but this is unlikely, as no SARS-CoV-2 antigen was detected in the explanted recipient’s heart upon immunohistochemical examination. In addition, the diagnosis of COVID-19 had also been ruled out before HTX via a PCR smear test. Perioperative infection is also unlikely. At the same time, reactivation of a SARS-CoV-2 infection is persistent in the endothelium of the donor heart. We found that the graft was SARS-CoV-2 positive in an electron-microscopic examination of an intraoperative biopsy from the left atrial fibrolipoma in the donor’s myocardium. Taken together, our observations suggested that SARS-CoV-2 was transmitted from the donor to the recipient in this case.

At the same time, there are research findings that transplanting hearts from COVID-19-positive donors can be safe and effective [22,23]. However, more information is needed regarding selecting COVID-19-positive donors.

The ability of SARS-CoV-2 to persist in the tissues and the duration of visual persistence remain unresolved questions. Long-term SARS-CoV-2 persistence in upper respiratory samples has been detected four to five months post-COVID-19 [24]. It is common knowledge that patients on immunosuppressive therapy were more susceptible to the prolonged persistence of SARS-CoV-2. The longest (over 8 months) viral shedding detected with a SARS-CoV-2-positive nasopharyngeal swab was documented in a patient with non-Hodgkin lymphoma [25]. Viremia in this patient was associated with COVID-19 relapse and was accompanied by a decrease in CD8+ T-lymphocyte count. A kidney transplant recipient also has been reported with prolonged (2 months) positive results of SARS-CoV-2 testing [26].

In our study, SARS-CoV-2 persisted in the myocardium up to 34 days after HTX. This conclusion was based on results of the third EMB (34 days post-HTX), where signs of endotheliitis were not found.

Mycophenolate mofetil and steroids can be effective for myocarditis treatment [27,28]. The other reason behind no detectable heart failure of the transplanted heart was the steroid management that was a part of the standard post-transplant immunosuppression protocol [29].

Electron-microscopic assessment of EMB in the post-HTX period revealed pericyte alteration. Pericytes were discovered in 1873 by the French scientist Charles-Marie Benjamin Rouget and were originally called Rouget cells [30]. Pericytes are found within the basement membrane of the capillary and surround the endothelial cells. Pericytes contact with endothelial cells by cytoplasmic processes and are considered to stabilize the vessel wall and control endothelial cell proliferation. Pericytes are present in a variety of tissue types, including lung, heart, and nervous tissue. There, they regulate blood flow, angiogenesis, and neovascularization; maintain the blood–brain barrier and tissue hemostasis; and participate in inflammation. The distinct feature of pericytes is their ability to differentiate into other cell types including adipocytes, chondrocytes, fibroblasts, vascular cells, and other cells of mesenchymal origin [31].

Vascular cells and immune cells circulating in the vascular system are known targets of different viruses. Moreover, the viral capacity to enter the blood flow and to produce viremia is crucial for viral spread. Results of several studies on cellular and organoid models clearly demonstrated that pericytes could be infected by SARS-CoV-2 [32,33]. However, the data from patients are scarce. Pericyte infection by SARS-CoV-2 has been seen in heart and brain tissue [34,35].

Angiotensin-converting enzyme 2 (ACE2) is a receptor enabling SARS-CoV-2 entry into the cell, and its expression was found in endothelial cells and pericytes [36]. However, endothelial cells express ACE2 at a very low level, while pericytes had the highest level of ACE2 expression [37]. This corroborates the idea that human pericytes are the key target for SARS-CoV-2 infection [38]. Pericyte injury leads to capillary endothelial cell dysfunction, thus causing microvascular dysfunction and microvasculopathy. Moreover, patients with cardiovascular conditions had an elevated level of ACE2 expression that increased the risk of a severe course of COVID-19 [39].

Under pathological conditions, cardiac pericytes can detach from the vascular wall and differentiate into myofibroblasts. This process results in increased vascular permeability and inflammation and ultimately contributes to interstitial fibrosis and destabilization of the vascular wall [40,41]. For that reason, cardiac pericytes can serve as a therapeutic target in the treatment of HF and other diseases [42].

Electron microscopy of EMB has demonstrated round particles with clear signs of morphological similarity to the SARS-CoV2, being associated with the apical surface of the endothelial cell. Fox SE et al. [43] also showed particles consistent with SARS-CoV-2 presented within a cardiac endothelial cell. On the other hand, reliable detection of SARS-CoV-2 using electron microscopy is challenging because of the structural similarity between the virion and intracellular structures [44]. We analyzed reported cases of coronavirus transmission during heart transplantation documented in the literature. Our aim was to examine the frequency and outcome of such transmissions. The results of our analysis are summarized in Table 2, which provides valuable information on the occurrence and consequences of SARS-CoV-2 transmission in the context of heart transplantation.

Based on the cumulative findings of previous studies, it can be inferred that in the majority of cases, heart transplantation was performed within 0–7 days after the last positive nasopharyngeal swab. However, PCR testing of myocardial tissue was conducted only in isolated instances. Similarly, post-transplantation endomyocardial biopsy (EMB) analysis for coronavirus antigens was carried out in only a few cases. Despite these limited data, none of the patients exhibited systemic coronavirus infection following heart transplantation.

Furthermore, the presence of coronavirus infection in the donor did not impact the outcomes of the recipients in any of the reported cases. Thus, heart transplantation from donors with coronavirus infection appears to be a relatively safe procedure rather than a risky one. Nevertheless, patients in such scenarios require more thorough postoperative monitoring to ensure their well-being.

## 4. Conclusions

The present report describes the unique case of SARS-CoV-2 transmission from donor to recipient during HTX, followed by activation of vascular wall cells (endothelial cells and capillary pericytes) and humoral immunity. We suggest examining all post-transplant EMBs to exclude SARS-CoV-2 transmission in the transplanted heart, especially in those with signs of humoral rejection.

## Figures and Tables

**Table 1:** Clinical data of the recipient.

Period of Time	Period of Treatment	Data of Examination
34th week of gestation	First complaints developed and patient was managed at home	- SARS-CoV-2 infection and had bilateral pneumonia - In 2 weeks the patient recovered from COVID-19 but was admitted to the local hospital due to clinical manifestation of heart failure
39th week of gestation	Patient moved to our Centre	- Patient gave birth to a healthy baby by cesarean section.
3 weeks after delivery	Heart failure ICU	- HFrEF, ECMO implantation - Included in the heart transplant waiting list
18 days after ECMO implantation	Heart failure ICU	Heart transplantation, ECMO explantation
8 days after heart transplantation	Heart transplantation clinical department	EMB results: acute cellular rejection (ACR (0R)) and antibody-mediated rejection (pAMR2 (I+, H+))
20 days after heart transplantation	Heart transplantation clinical department	EMB results: signs of humoral rejection (pAMR2 (I+, H+))
34 days after heart transplantation		EMB results: ACR (1R/1A) and pAMR0

Abbreviations: HFrEF—heart failure with reduced ejection fraction; ICU—intensive care unit; ECMO—extracorporeal membrane oxygenation; EMB—endomyocardial biopsy; ACR—acute cellular rejection; AMR—antibody-mediated rejection.

**Table 2:** Analysis of reported cases of coronavirus transmission during heart transplantation.

Authors	Cases	Proof of Coronavirus in a Donor	Proof of Coronavirus in a Recipient	Complication
Shin Lin et al. [45]	5 cases of heart transplantation from COVID-19-positive donors	NP RT-PCR: positive (0–1 days prior) Bronchoalveolar lavage PCR test—positive (0 days) Blood IgG spike—positive (0 days)	None	1 case—primary graft dysfunction 4 cases—none or nonspecific complication
Jashari R. et al. [46]	2 cases of heart transplantation from COVID-19-positive donors	NP RT-PCR: positive (0 days prior) PCR test on the myocardium and heart valves—negative	PCR of the cardio-vascular tissues—negative	None
Castro-Varela A. et al. [47]	6 cases of heart transplantation from COVID-19-positive donors	NP RT-PCR: positive (1–7 days prior)	NP RT-PCR: negative	3—primary graft dysfunction 1—diffuse myocyte injury, pericarditis, death
Eichenberger E.M. et al. [48]	9 cases of heart transplantation from COVID-19-positive donors	NP RT-PCR: positive (1–7 days prior)	NP RT-PCR: negative	1—detection of coronavirus antigens in the myocardium using IHC 1—massive hemorrhage and coronary thrombosis
Vaidya, G. N. et al. [49]	177 cases of heart transplantation from COVID-19-positive donors	NP RT-PCR: positive (0–14 days prior)	NP RT-PCR: negative	6—primary graft dysfunction 9—death
Neidlinger N. A. et al. [10]	1 cases of heart transplantation from COVID-19-positive donors	Stool PCR positive (3 days prior) NP RT-PCR: negative (0 days prior)	NP RT-PCR: negative	None
Madan S. et al. [50]	150 cases of heart transplantation from COVID-19-positive donors	NP RT-PCR: negative (0–2 days prior)	NP RT-PCR: negative	23—mortality, cumulative Kaplan–Meier estimated primary graft dysfunction—no data

Abbreviations: NP RT-PCR—nasopharyngeal swab reverse transcription PCR.

## Data Availability

If you need clarifications, or need additional information, you can write to the email: doctormakarovia@gmail.com.

## Abbreviations

ACR	acute cellular rejection
AMR	antibody-mediated rejection
CD	cluster of differentiation
EF	ejection fraction
EMB	endomyocardial biopsy
ECMO	extracorporeal membrane oxygenation
HF	heart failure
HLA-DR	Human Leukocyte Antigen DR isotype
HHV	Human Herpes Virus
HTX	heart transplantation
LV	left ventricle
PCR	polymerase chain reaction
RNA	ribonucleic acid
VEGF	vascular endothelial growth factors
VWF	von Willebrand factor
